# Iodine an Antidote to the Venom of the Rattlesnake

**Published:** 1860-05

**Authors:** James S. Whitmire

**Affiliations:** Metamora, Ill.


### IODINE

### AN ANTIDOTE TO THE VENOM OF THE RATTLESNAKE, CROTALUS, AND IN THE TREATMENT OF THE BITE.

BY JAMES S. WHITMIRE, OF METAMORA, ILL.

In the December, or January number of the *N. W. Medical and Surgical Journal*, of 1849, or '50, I published an article in relation to the tinct. of iodine in the treatment of the bite of the rattlesnake; in which I argued that the venom produced debility of the capillary vessels, first by direct contact, and afterwards, by its absorption into the general circulation, it effected the entire system; and that the tinct., used as a paint upon the part, gave tone to the vessels by being absorbed; and that I consequently considered it a rational treatment. I suggested, in the same article, that iodine was an antidote to the venom, and believed it to be so, on account of the extraordinarily short time in which the system seemed to get rid of the poison, and ceased to be disturbed by its prostrating effects, especially, when the remedy was used immediately, or even within six hours of the insertion of the venom. This was written after having treated many cases, during the years 1845, '46, '47, '48 and '49; and I had become thoroughly convinced, that the treatment of the bite, of all venomous serpents or reptiles, by iodine, was altogether preferable to any other then known to the profession. At that time, I had not satisfied myself, by actual experiments, that it was an antidote to the venom. Soon after this, however, Professor Brainard, of Rush Medical College, instituted a series of experiments, which were duly reported to the Illinois State Medical Society, at one of its subsequent sittings, in an ably written paper, read before the Society, proving, beyond a doubt, that iodine wrs a perfect antidote to the poison, completely destroying its effects on the animal economy, and therefore an indespensible agent in the treatment of the bite. Since his experiments, I have repeated them for my own satisfaction, with the same results. I have since then, again made some experiments of a different and interesting character, which, even though they prove of no practical value, will, nevertheless, go far to establish a still mooted point; viz: whether the venom, when swallowed, will produce the same constitutional effects that are developed by it when injected into the celluar tissue by the bite. For this purpose I procured three of the largest, of the kind, that I conld find on the prairie, and decapitated them, and proceeded to seperate the poison-gland, sac that contains the secretion, and fang, all together, from under the orbit. By pressing the gland and sac between the thumb and finger, I could get from one to two drops from the canal in the fang, which I washed off in sweet milk, (I used six fangs in these experiments,) and gave it to two young dogs, four months old, and three cats about the same age. To one dog and one cat, by means of a stomach tube, I gave each iii gr. of iodine and vi gr. of iod. pottass rubbed up in water. In about two hours they both began to be restless and uneasy and finally began to be languid and feverish and would take no nourishment, but would take water when offered to them. In six hours after, I repeated the dose; and in twenty-four hours they began to assume their wanted spirits, and never showed any distinguishable signs of poisoning. The other dog, and one cat, I did nothing for, and in about an hour they both began to show signs of illness. The cat mewed and gave out the most piteous cries, and seemed to be in the most excruciating agony, had fever and great thirst, drinking quantities of water, which it would eject from its stomach in twenty or thirty minutes. In five hours it began to swell and purge, so that, in fifteen hours the whole cellular tissue was infiltrated with serum. After this it had sanguineous discharges from the bowels; and at the end of twenty-four hours it died in convulsions.

The dog was held in very much the same condition, excepting when water was placed before him he would rise up on his fore legs and lap it greedily, and then sink back into the same languid condition. He vomited and seemed exceedingly prostrated for ten hours, and then remained stupid and whined, as if in pain, for twenty-four hours, after which he began to have bloody discharges from the bowels. He commenced swelling about the tenth hour, and at this time he began to have a thin sero-sanguinous fluid run from his eyes, and the vessels of the conjunctiva were greatly engorged, and the cellular tissues under the conjunctiva was so infiltrated with bloody serum that his eyes had more the appearance of balls of clotted blood than orbs of vision. He remained in this condition for about five days, taking no nourishment, but drinking a great quantity of water, though this was attended with difficulty, on account of the swollen condition of his tongue. He now began to show signs of improvement, recovering his appetite slowly, his eyes losing their clotted appearance, and the swelling to cease; so that in six days Ponto was himself again.

To the last cat I gave diluted alcohol, every four hours, so soon as it began to show signs of illness, which commenced with the same languor, lassitude, pain and fever as the others did. It wanted no food, but drank considerable water, being stupid all the time, either from the effects of the alcohol, or the poison. It remained stupid and feverish for three days, after which it began to recover; and in about ten days I discharged my patient as cured, though it was still weak, and would have been the better of longer care.

My impression now is, in regard to the popular alcoholic treatment, that it does not act as an antidote to the venom, but merely stimulates the nervous system, through the great centers, and keeps the system above the depressing influence of the poison, giving nature a chance to get rid of the poison through its own resources.

These experiments have convinced me, that the venom, whether swallowed, or injected into the cellular tissue, produces precisely the same results upon the animal economy, only the former acts more generally and the latter locally.—Since I commenced the use of iodine, in the treatment of the bite of the rattlesnake, I have had every reason to be satisfied with its use; never, in the course of fourteen years' country practice, having been disappointed in its effects. During that time I have treated 75 cases in man, which have comprised every grade of the effects of the poison that may be produced, reasonably, short of absolute dissolution, to within an hour of the infliction of the wound by the serpent. When called in a recent case, or even within fifteen hours of the bite, I now adopt Professor Brainard's plan; which is, to inject the cellular tissue, in and about the wound, with the tinct. of iodine, by means of a sharp pointed silver syringe, and then proceed to paint the swollen parts thoroughly with the tinct., and try to keep in advance of the swelling, from two to three inches; and in recent cases this is all the treatment necessary to complete the cure.

If called after the constitutional effects of the poison have become developed, I paint the limb, and even the whole boby, with the tinct., and give, internally, wine or brandy, with iod. potass. and chlor. potass. dissolved and largely diluted in water, till the urgent symptoms begin to subside, and then administer quinine and iron as a tonic, &c. I have treated two extreme cases, it having been three days from the time of the bite till I was called. In both cases there was fever and delirium, with extreme prostration, the wound black with infiltrated blood and vesicated patches on the limb filled with a sero-sanguinous fluid, and hemoragic discharges from the bowels. For the latter, I prescribed solid opium, and a turpentine emulsion, with kreosote, so that my patient got one drop of the latter every four hours, at a dose. I gave them chlorate potash and iod. potass. dissolved and largely diluted with water, and brandy or wine, ad. libitum. I painted my patients nearly from head to foot with the tinct., and varied my treatment according to the urgencies that presented till convalescence was established, which was in about ten days from the bite. Their convalescence were slow and tedious but finally they recovered their usual health, strength and spirits.

